# Development of a Novel *ex vivo* Nasal Epithelial Cell Model Supporting Colonization With Human Nasal Microbiota

**DOI:** 10.3389/fcimb.2019.00165

**Published:** 2019-05-21

**Authors:** Derald D. Charles, James R. Fisher, Sarah M. Hoskinson, Audrie A. Medina-Colorado, Yi C. Shen, Mohamad R. Chaaban, Steven G. Widen, Tonyia D. Eaves-Pyles, Carrie A. Maxwell, Aaron L. Miller, Vsevolod L. Popov, Richard B. Pyles

**Affiliations:** ^1^School of Medicine, University of Texas Medical Branch, Galveston, TX, United States; ^2^Department of Microbiology and Immunology, University of Texas Medical Branch, Galveston, TX, United States; ^3^Department of Pediatrics, University of Texas Medical Branch, Galveston, TX, United States; ^4^Department of Otolaryngology, University of Texas Medical Branch, Galveston, TX, United States; ^5^Department of Biochemistry and Molecular Biology, University of Texas Medical Branch, Galveston, TX, United States; ^6^Department of Pathology, University of Texas Medical Branch, Galveston, TX, United States

**Keywords:** nasal microbiome, nasal mucosa, culture model, respiratory system, human

## Abstract

The nasal mucosa provides first line defense against inhaled pathogens while creating a unique microenvironment for bacterial communities. Studying the impact of microbiota in the nasal cavity has been difficult due to limitations with current models including explant cultures, primary cells, or neoplastic cell lines. Most notably, none have been shown to support reproducible colonization by bacterial communities from human donors. Therefore, to conduct controlled studies of the human nasal ecosystem, we have developed a novel *ex vivo* mucosal model that supports bacterial colonization of a cultured host mucosa created by immortalized human nasal epithelial cells (NEC). For this model, immortalized NEC established from 5 male and 5 female donors were cultured with an air-interfaced, apical surface on a porous transwell membrane. NEC were grown from nasal turbinate tissues harvested from willed bodies or from discarded tissue collected during sinonasal procedures. Immortalized cells were evaluated through molecular verification of cell type, histological confirmation of tissue differentiation including formation of tight junctions, NEC multilayer viability, metabolism, physiology and imaging of the luminal surface by scanning electron microscopy. Results showed proper differentiation and multilayer formation at seven to 10 days after air interface that was maintained for up to 3 weeks. The optimized mucosal cultures created an environment necessary to sustain colonization by nasal microbiomes (NMBs) that were collected from healthy volunteers, cryogenically preserved and characterized with customized quantitative polymerase chain reaction (qPCR) arrays. Polymicrobial communities of nasal bacteria associated with healthy and inflamed states were consistently reproduced in matured NEC co-cultures by transplant of NMBs from multiple community types. The cultured NMBs were stable after an initial period of bacterial replication and equilibration. This novel *ex vivo* culture system is the first model that supports controlled cultivation of NMBs, allowing for lab-based causation studies and further experimentation to explore the complexities of host-microbe and microbe-microbe interactions.

## Introduction

While filtering, warming, and humidifying inspired air, the human nasal cavities provide first line immunologic defenses and create a unique microenvironment supporting colonization by commensal microbiota distinct from other body sites (Proctor and Relman, 2017). The epithelial composition of the anterior nares is most similar to external skin: keratinized stratified squamous epithelium including hairs, sweat glands, and sebaceous glands. Moving deeper into the nasal vestibule, the mucosa is a non-keratinized, stratified squamous epithelium that transitions to pseudostratified columnar epithelium typical of the nasal cavities (Jafek, 1983). In addition to its physical tight junction barriers, respiratory epithelial mucus traps smaller particulates and bacteria for disposal by mucociliary action (Hariri and Cohen, 2016). Numerous cell surface pathogen-recognition receptors are produced by the nasal mucosa including toll-like receptors, nucleotide-binding oligomerization domain-like receptors, and retinoic acid-inducible genes that perform immunomodulatory functions (Schleimer et al., 2007). Actions of activated pathogen-recognition receptors include signaled release of a cocktail of antimicrobial peptides into nasal mucus (antimicrobial enzymes, permeabilizing peptides, protease inhibitors, and neutralizing proteins) and the release of leukocyte-recruiting cytokines (Schleimer et al., 2007).

Current options for nasal tissue models include organotypic explant culture, primary cell culture, and culture of various neoplastic cell lines. Organotypic explant culture techniques provide the closest approximations of human nasal tissue *in situ*, but use of these models for causation studies is limited due to a lack of standardization. Despite having proper tissue architecture and differentiation, acquiring suitably sized explants can be difficult and unreliable. Additionally, reproducibility of organotypic explants proves challenging due to genetic variability and uncontrollable environmental variables prior to tissue harvest (Pezzulo et al., 2011). Culturing primary NEC with an air-liquid interface (ALI) improved upon organotypic explant models in that harvested tissue may be expanded into multiple specimens prior to culture studies (de Borja Callejas et al., 2014). NEC in liquid cell culture (LCC) expand as a monolayer of simple cuboidal cells, but when seeded and grown at an ALI, the cells differentiate into nasal epithelial tissue multilayers with comparable architecture, mRNA profiles and cellular immune responses to tissue (Bücheler and Haisch, 2003; Pezzulo et al., 2011; de Borja Callejas et al., 2014). Although improved, primary cell culture can pose challenges due to limits on the number of times the cultures can be passaged before senescence. This necessitates repeated collection of tissue from donors which can introduce biologic variability due to genetic differences or temporal variability in sample collection (Cho et al., 2010; Haghi et al., 2014; Papazian et al., 2016).

Finally, immortalized nasal and respiratory cell lines are available, such as the human nasal squamous cell carcinoma derived RPMI2650, the SV40-transformed bronchial cell line 16HBE14o- and the human lung adenocarcinoma derived Calu-3, that provide ample standardized and reproducible respiratory cell cultures. Unfortunately, these cell lines exhibit genetic and phenotypic discrepancies compared to primary cells, limiting *in situ* applicability of results (Cozens et al., 1994; Florea et al., 2003; Cho et al., 2010; Haghi et al., 2014; Papazian et al., 2016). Further, these cells fail to effectively model the nasal mucosa as they are primarily derived from deeper respiratory tract tissues. Despite the differences, utilization of immortalized respiratory cell cultures with conserved physiologic characteristics of interest proves useful in predicting *in situ* outcomes (Florea et al., 2003; Cho et al., 2010; Haghi et al., 2014; Papazian et al., 2016).

High-throughput sequencing technology created a cultivation-independent means to survey colonized commensal nasal bacteria in large cohorts, however, an understanding of relationships between commensal nasal microbiota and disease requires additional elucidation (Consortium, 2012; Liu et al., 2015). Microbial communities can be quite diverse between individuals, but bacteria with an affinity for nasal colonization capable of predominating the microbiota have been utilized to establish 7 nasal microbiome community-state types (CSTs). These include members of the *Staphylococcus* genus, especially *S. aureus* (CST1) *and S. epidermidis* (CST3); *Enterobacteriaceae* (CST2), the *Propionibacterium* genus (CST4), including *P. acnes* and *P. granulosum*; *Corynebacterium spp*. (CST5), *Moraxella spp*. (CST6), *and Dolosigranulum spp*. (CST7) (Lemon et al., 2010; Ramakrishnan et al., 2013; Liu et al., 2015). Correlations between nasal microbial colonization and health outcomes are well established clinically due to the common colonization of nasal cavities by various pathobionts. *S. aureus* (prevalence of 30%) is a prime example with the associated increased incidence of infection by the pathogen (Wertheim et al., 2005; Bode et al., 2010). Nasal pathobionts exist in complex microbial communities and recent in-depth studies of these communities has identified interspecies interactions as a novel source of clinically relevant information and potential opportunities for novel interventions (Bomar et al., 2016; Zipperer et al., 2016).

Although extensive surveys of microbes colonizing the human nasal cavity have provided correlations between health outcomes and nasally residing commensal organisms, they have been unable to systematically test the community in controlled models. As such, we sought to create a system for culturing microbiota as communities in the context of the human host tissue to support NMB causation studies. In this study we characterized an immortalized NEC model optimized for laboratory standardization of both the human tissue *ex vivo* microenvironment and NMB bacteria inoculum. When cultured with an ALI in either 24 or 96 transwell formats, the resulting NEC multilayer maintained its epithelial barrier integrity while also supporting controlled colonization by communities of diverse nasal bacteria representing selected community types (Liu et al., 2015). This model system will prove useful for future controlled causation studies of the impact of polymicrobial colonization on cultured NEC mucosae to help elucidate host-microbe and microbe-microbe interactions and for use in preclinical screening of probiotics and therapeutics.

## Materials and Methods

### Ethics Statement

Collection of NMB from healthy volunteers was approved by the institutional review board at University of Texas Medical Branch under the protocol number 17-0018. Adults, 18 years and older filled out a brief questionnaire and provided written consent before being assigned a unique study number. Each participant was instructed on sample collection and observed during the nasal swabbing. De-identified, discarded tissue was collected from sinonasal procedures performed by the University of Texas Medical Branch Otolaryngology Department or from nasal tissues collected from willed bodies through an approved research protocol with the National Disease Research Interchange (NDRI; Philadelphia, PA, USA).

### NEC Isolation, Immortalization and Culture

We established primary NEC cultures from nasal tissues using an optimized epithelial cell outgrowth procedure (Herbst-Kralovetz et al., 2008). All experiments involving cell culture and bacterial culture were conducted under BSL-2 conditions. Most of the male cultures were obtained through the NDRI service. Discarded turbinate tissues from sinonasal procedures performed by the UTMB Otolaryngology service also were collected and utilized to provide additional male and female cultures as shown in Table 1. Nasal tissues were processed within 48 h of collection after being transported to the lab at 4°C in Dulbecco's Modified Eagle Media (DMEM; ThermoFisher, Waltham, MA, USA) with heat-inactivated 10% newborn calf serum (ThermoFisher) and standard antibiotic and antimycotic (10,000 units/mL of penicillin, 10,000 μg/mL of streptomycin, and 25 μg/mL of Amphotericin B; ThermoFisher).

**Table 1:** Nasal epithelial cell cultures created for this study.

**Immortalized NEC culture**	**Date**	**Donor**	**Haplogroup**	**Molecular NEC confirmed**	**Culture status**
NEC01	Apr, 2015	Caucasian/M/52/ABM primary cell culture	T2b	No	Discontinued
NEC02	Aug, 2015	Black/M/46 COD MI	L3	Yes	Active
NEC03	Nov, 2015	Caucasian/M/69 COD Trauma	K	Yes	Active
NEC04	May, 2017	Caucasian/M/32 COD Septic shock/DMID	H	Yes	Lost due to contamination consistent with donor COD
NEC05	June, 2017	Black/M/46 COD AAA	R8a1	Yes	Active
NEC06	June, 2017	Caucasian/M/66 COD Alzheimer's	H	Yes	Active
NEC07	Aug, 2017	Caucasian/M/70 COD Myocardial Infarction	H	Yes	Active
NEC08	Oct, 2017	Caucasian/F turbinate reduction	H	Yes	Active
NEC09	Nov, 2017	Caucasian/F/64 COD ALS	T2b	Yes	Active
NEC10	Dec, 2017	Caucasian/F/72 Mucosal hypertrophy	T2	Yes	Active
NEC11	Dec, 2017	Black/F/18 allergic fungal sinusitis	N/A	Yes	Lost due to fungal contamination consistent with donor cause of surgery
NEC12	Dec, 2017	Caucasian/F/33 Allergic rhinosinusitis	D	Yes	Active
NEC13	Apr, 2017	F	L2	Yes	Active

*Molecular analysis of immortalized NEC. NEC01 culture was commercially purchased, but did not pass our verification process. NEC04 and NEC11 were discontinued due to contamination. All other cultures are active, passed verification, and were haplotyped in concordance with the donor*.

To prepare the tissues for epithelial outgrowth, they were washed with 1X Dulbecco's phosphate buffered saline (DPBS; Corning, Corning, NY, USA) prior to submersion in 0.25% trypsin/EDTA and incubation at 37°C. Every 10 min, the samples were agitated, until evidence of digestion was visible. Trypsin incubation times ranged from 10 to 30 min. Enzymatic digestion was halted by addition of 40 mL of neutralizing medium (DMEM with 10% serum and antimicrobials as noted above) before centrifugation at 1,500 RPM for 5 min. A portion of the decanted supernatant was used to create a DNA lysate sample for subsequent analysis. The digested tissues were placed into a 100 mm petri dish where cartilaginous tissue was removed and the mucosa was processed into small 3 mm^3^ pieces. Each tissue piece was cultured in keratinocyte serum free medium (KSFM; Invitrogen, Carlsbad, CA, USA) supplemented with 5 ng/mL of recombinant epidermal growth factor, 50 ug/mL bovine pituitary extract, 44.5 ug/mL of CaCl_2_, and 0.2 mg/mL of primocin (InvivoGen, San Diego, CA, USA). NEC outgrowth was observed daily and, when confluence was observed near tissue masses, the cells were collected by trypsinization and plated in fresh culture vessels followed by HPV E6/E7 immortalization as previously described (Halbert et al., 1992; Rose et al., 2012).

All primary and immortalized cells were propagated in KSFM at 37°C with 5% CO_2_ supplemented air. Cultures were refed every other day and tested for mycoplasma contamination monthly (MycoSensor qPCR assay, Agilent, Santa Clara, CA, USA). Passaging occurred every 5–7 days when 80% confluency was reached. ALI cultures were produced 24–48 h after plating optimized numbers of NEC in KSFM without primocin to apical chambers of 24 or 96 well format transwell inserts (Greiner Bio-One, Monroe, NC, USA). KSFM without primocin was replaced in the basal chamber of all cultures and the apical chamber of liquid-liquid interfaced (LLI) cultures every other day.

### Molecular Verification of NEC Phenotype and Donor Haplotyping

Each sample consisting of at least 10^6^ NEC were lysed in 100–200 ul of MagNAPure External Lysis Buffer (Roche, Indianapolis, IN, USA) for extraction using MagNAPure 96 Cellular RNA large volume kit (Roche) and converted to cDNA (iScript, Bio-Rad, Hercules, CA, USA) immediately or, for DNA, using the MagNAPure 96 DNA Viral NA small volume kit (Roche). Verification of the gender, epithelial and nasal origin of the immortalized NEC was completed using a customized qRT-PCR array to detect expression of selected genes. Targets included genes expressed by human nasal mucosae [nasal (BPIFA1), oral (STATH), epithelial cells (EpCAM)] (Juusola and Ballantyne, 2005; Lindenbergh et al., 2012; Hanson and Ballantyne, 2013; Xu et al., 2014; van den Berge et al., 2016; van den Berge and Sijen, 2017), X and Y chromosomes (Male = RPS4Y1, Female = XIST) and a housekeeping gene (PSMB2) for quality assurance of the RNA preparation. Qualified NEC cultures (shown in Table 1) were expanded and cryopreserved at low passages. Phenotypes, transcription profiles and general morphology were indistinguishable across at least 40 passages when fresh cells were thawed.

DNA samples from each NEC donor and subsequently established culture also were haplotyped using a custom qPCR array followed by pyrosequencing of the amplimers on a PyroMark 96 pyrosequencing system (Qiagen, Germantown, MD, USA). Briefly, template DNA was subjected to 16 individual PCR-based pyrosequencing reactions with subsequent pyrosequencing using PyroMark Gold Reagents (Qiagen). PCR primers targeted both haplogroup-designating SNPs and hypervariable regions I and II in mitochondrial DNA (Andréasson et al., 2002; Guseva et al., 2007; Salas and Amigo, 2010; Johnson et al., 2015). SNPs were compared to the rCRS reference sequence for designation of known haplogroups. Hypervariable sequences were aligned to the rCRS reference sequence with the resulting polymorphism patterns compared to the reference database mtDNA manager further defining haplogroups or unique sequence between samples (Lee et al., 2008). Haplotypes also served to confirm the identity and purity of passaged cultures periodically throughout culturing and experimentation.

### Characterization of Transwell NEC Cultures

Transepithelial electrical resistance (TEER) measurements were recorded for triplicates of each NEC culture in 24 transwell format on days 3, 7, 14, and 21. TEER readings were measured using an EVOM^2^ Voltohmmeter outfitted with chopstick electrodes (World Precision Instruments, Sarasota, FL, USA) after covering the apical surface of ALI transwell cultures with 100 μL DPBS. Normalization of triplicate values involved multiplying the measured resistance by transwell membrane surface area (0.33 cm^2^) to be compared against transwell controls that did not contain multilayer cell formations.

For histological examination, NEC tissues were fixed in Z-fix (Anatech, Battle Creek, MI, USA) for 1 h at 4°C on day 7 after plating for LLI cultures and on days 3, 7, 14, and 21 for ALI conditions. The fixed membranes were excised from the cup and paraffin embedded before 5 μm sectioning with a Microm HM 310 microtome (ThermoFisher). Non-sequential sections (*n* = 4 per sample) of replicate cultures (*n* = 3) were mounted and then stained with hematoxylin and eosin (H&E; VWR, Radnor, PA, USA) and imaged at 20X or 40X on an EVOS imaging system (Life Technologies, Carlsbad, CA, USA).

To confirm that NEC in transwell cultures produced relevant mucins, we compared the mucin profile to that from NEC in monolayer format via dot blot assays as previously described (Harrop et al., 2012). Protein (20 ug) from 80% confluent monolayer cultures and 4 and 8 day old ALI cultures was collected by lysis in RIPA lysis buffer (Sigma-Aldrich; St. Louis, MO, USA) supplemented with 200 ug PMSF and placed in dots on nitrocellulose paper. The dot blots were stained with 0.1% Ponceau S (Sigma-Aldrich) to confirm protein binding, approximate quantity and location before each blot was blocked in 5% non-fat dry milk in TBS. Individual blots then were incubated overnight at 4°C with primary murine antibodies to MUC1, MUC2, MUC4, MUC5AC, MUC5B, or Mel-CAM diluted as recommended by the supplier (Santa Cruz Biotechnology, Santa Cruz, CA, USA). After washing in TBS with 1% Tween-20 the blots were incubated for 4 h at room temperature with a 1:500 diluted goat anti-mouse polyclonal antibody conjugated to horseradish peroxidase (Seracare, Milford, MA, USA). After extensive washing, the blots were developed with Pierce SuperSignal West Pico Chemiluminescent Substrate (ThermoFisher) and exposed to x-ray film (Kodak, Rochester, NY, USA) for 3 min.

To compare transcriptional profiles between primary and immortalized NEC cultures, RNA-seq was performed on parallel samples of RNA from representative male and female cultures. Briefly, for these studies, 24 well transwells were seeded with 1 x 10^5^ primary or immortalized NECs in parallel, air-interfaced, and allowed to mature for 7 days. At 7 days, 100 ul MagNApure External lysis buffer was used to lyse the cultures and support automated RNA recovery. The NEBNext Ultra II RNA library kit (New England Biolabs, Ipswich, MA, USA) was used to prepare the RNA-Seq library from poly(A) selected RNA. Paired-end 75 base sequencing was performed on a NextSeq550 by the NGS core facility, University of Texas Medical Branch, Galveston, Texas. Reads were mapped using STAR version 2.6.1c, using the GRCh38 reference genome and the Gencode V28 annotation file. Read counts from the STAR –quantMode GeneCounts option were input into the R (Version 3.5.1) package DESeq2 (Version 1.22.2) for differential gene expression analysis.

### NMB Collection, Transplant to NEC and Community Profile Analysis by Quantitative Polymerase Chain Reaction (qPCR)

After obtaining informed consent, participants swabbed each nare with a sterile calcium alginate swab (ThermoFisher) avoiding contact with external sites. Swabs were placed into a tube containing 2 mL of sterile DPBS placed on ice and then processed within 30 min of collection. Processing began with a 10 s vortex before sterile creation of cryopreserved viable communities (aliquots were supplemented with 10% sterile glycerol) and additional 100 ul aliquots created for subsequent DNA, RNA, proteomic and metabolomic analyses. The DNA and RNA aliquots were mixed with an equal volume of MagNAPure External Lysis Buffer (Roche) to support magnetic particle-based extraction as described above. The DNA and RNA aliquots were mixed with an equal volume of MagNAPure External Lysis Buffer (Roche) to support magnetic particle-based extraction as described above while stabilizing DNA and RNA lysates. All of the aliquots were stored at −80°C.

Molecular characterization of the community profile was performed via our NMB custom qPCR array (See Supplementary Table 1) created as described previously (Veselenak et al., 2015). With optimized thermocycling protocols and primer designs, this array included 46 bacterial targets most commonly found in the nasal cavity and a universal 16S target to quantify total bacteria genomes and a GAPDH target to measure human genomic loads. For NMB transplantation studies, matured NEC 96 well transwell cultures (7–14 days post ALI), were inoculated with selected cryopreserved NMB that were thawed on ice and diluted to a final target concentration of 1 × 10^5^ bacterial genome equivalents per 10 ul inoculum that was established as optimal for creating consistent colonization without losing minor community members. Prepared 10 ul inocula were gently delivered onto the apical surface of matured NEC cultures and incubated at 37°C in a humidified chamber with 5% CO_2_ supplemented air. Triplicate wells were harvested for each condition at 24, 48, and 72 h post NMB transplantation by adding 200 ul of MagNAPure External Lysis Buffer (Roche) to assess colonization outcomes. The extracted DNA then was analyzed by custom PCR array and selected single target qPCR.

### Scanning Electron Microscopy (SEM) for Visualization of NMB and Single Species Colonization

Sterile, NMB communities- or methicillin-resistant *Staphylococcus aureus* (MRSA)-colonized NEC cultures in 24 well transwell cups (*n* = 3–4 for each condition) were fixed 48 h after bacterial application in primary fixative solution (1.25% paraformaldehyde, 2.5% glutaraldehyde, 0.03% CaCl_2_,50 mM cacodylate buffer) and stored at 4°C. The MRSA strain USA300 isolated in our lab from a pediatric nasal sample was cultivated in Tryptic Soy broth overnight at 37°C with agitation, and 5 × 10^2^ bacteria were added to the NEC culture. Each sample was processed by rinsing in 0.1 M cacodylate buffer, postfixed in 1% OsO_4_ in 0.1 M cacodylate buffer and then dehydrated in absolute ethanol and hexamethyldisalazane. After air drying, the membrane was removed, mounted for sputter-coating with iridium in an Emitech K575 × Sputter Coater (Emitech, Houston, TX, USA) at 20 mA for 20 s and then examined as described previously (Rose et al., 2012). Imaging was completed on a Hitachi S4700 SEM (Hitachi High Technologies, Schaumburg, IL, USA) with indicated magnifications.

## Results

### Immortalized Human NEC Cultured in Transwells Differentiated Into Mucosal Multilayers

**Figure 1:**
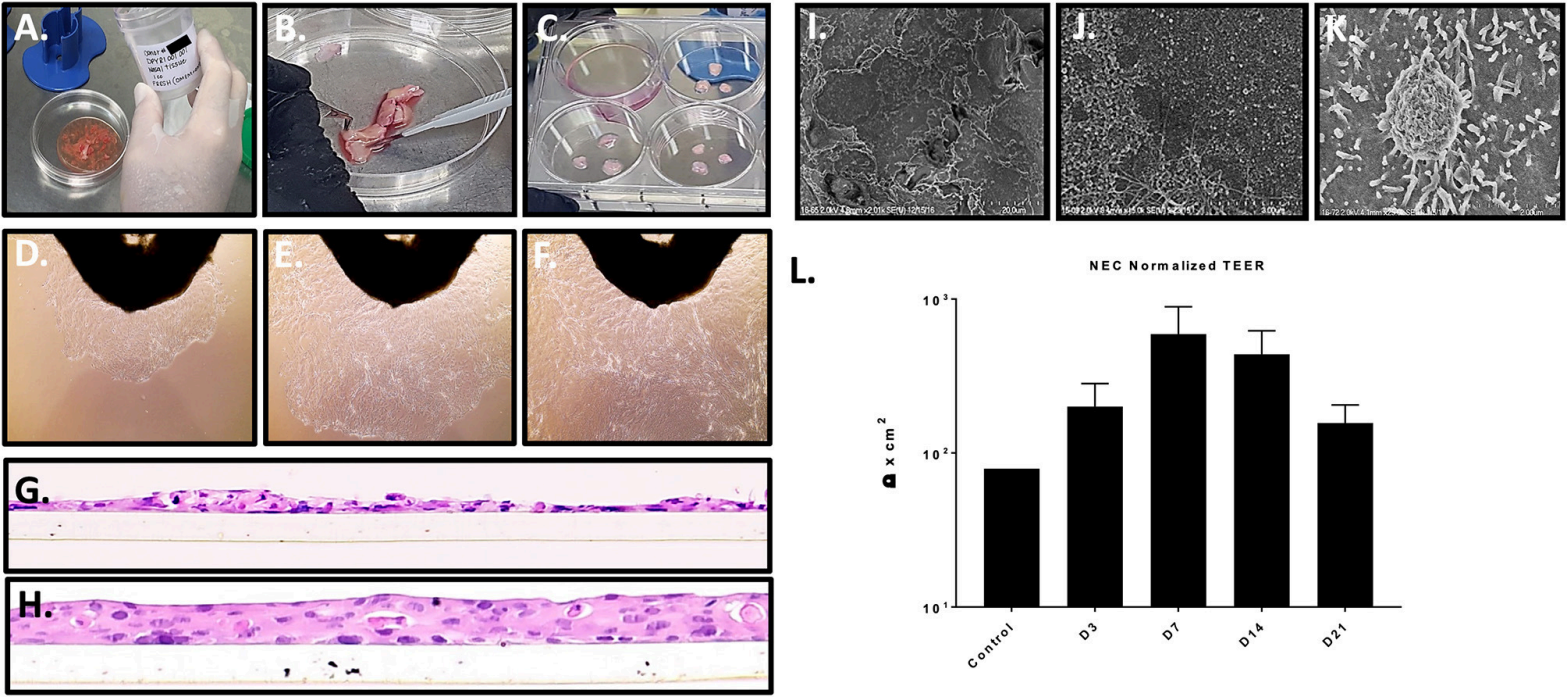
Nasal tissue processing, immortalization, and model generation. **(A–C)** Processing primary nasal tissue for immortalization. **(D–F)** Nasal cell outgrowth from primary tissue (observed at top of frame) used for immortalization (20X magnification). (**G,H)** staining of cross sections of LLI cultures of immortalized NEC revealed a lack of differentiation and monolayer formation **(H)** (20X magnification). The transwell membrane can be seen beneath the cells. ALI culture of immortalized NEC led to differentiation and multilayer formation, closely resembling native tissue architecture. These studies were completed with similar results across 4 distinct NEC. **(I–K)** The apical surface of differentiated NEC visualized by SEM revealed microvilli and mucin vacuoles. **(L)** TEER values peaked by 7 days, indicating epithelial barrier integrity as shown by average values across the NEC cultures (*n* = 3 replicates for each NEC culture). SEM magnification is shown at 2 k **(I)**, 15 k **(J)**, and 25 k **(K)**.

We acquired 14 different primary nasal mucosa tissue samples: 1 commercial cell culture, 7 samples supplied by NDRI from the turbinates of cadaveric donors and 7 samples from sinonasal procedures performed by the UTMB Otolaryngology service. After processing primary nasal tissue (Figures 1A–C), material from 10 donors (NEC02, 03, 05, 06, 07, 08, 09, 10, 12, 13) produced optimal cell outgrowth for subsequent immortalization and characterization (Figures 1D–F). Successful immortalization was indicated by passage to a third culture vessel when primary cells had reached senescence. Immortalized NEC were collected at passage 4 and molecularly analyzed to confirm they were of nasal and epithelial origin and that they matched the haplotype and gender of the donor material (Table 1). Through these analyses, the commercial cell culture, NEC01, was eliminated from the panel because the cells did not express the nasal marker BPIFA1 nor did they express the expected gender markers. NEC04 was discontinued due to culture contamination consistent with the donor's cause of death and NEC11 was discontinued due to a persistent fungal contamination consistent with the donor's health history. The remaining 10 cultures were molecularly confirmed to be of nasal tissue origin and together represent 8 different haplogroups (Table 1). All 10 cultures were successfully expanded and cryopreserved with extensive repeat testing to confirm purity and identity as well as testing to confirm they were free of any contaminating mycoplasmas.

Immortalized NEC from 4 distinct donors were subsequently cultured in transwell inserts at both liquid-liquid interface (LLI) and air-liquid interface (ALI). As expected, based on prior experience with similar model systems, LLI NEC in transwell format failed to differentiate (Figure 1G) establishing predominately single cell layers similar to the cobblestone monolayers of NECs grown in flasks. In cross section, H&E histology of paraffin sections of LLI NEC revealed occasional mounds of double and triple-cell layers (Figure 1G). In contrast to NEC cultured at LLI, each of the immortalized NEC cultures reliably differentiated into confluent tissue multilayers of 3 to 4 nucleated cells when cultured at ALI (Figure 1H).

Matured NEC multilayers from each donor exhibited epithelial morphologies comparable to those of primary cells cultured at ALI, including stratified cuboidal and stratified columnar cells situated onto a basal progenitor cell layer. None of the immortalized NEC fully recreated ciliated cells seen in primary ALI cultures (Müller et al., 2013; Park et al., 2016) but this also may be due to differences in the culture medium employed. Importantly, H&E histology of NEC cultures created across multiple passages revealed consistent tissue structure indicating reproducibility of the model system up to at least passage 40. H&E specimens evaluated throughout multilayer differentiation (days 1–28 post ALI) illustrated terminal multilayer thickness at 21 days to be 4–5 cells confirming the immortalized NEC cells still exhibit contact inhibition. Scanning electron microscopy (SEM) of the apical surfaces was completed for each NEC culture in matured multilayers fixed at 14 days post ALI and illustrated numerous apical cells coated in microvilli (Figures 1I–K) as well as structures consistent with mucin vacuoles. Trans-epithelial electrical resistance (TEER) was used to quantify polarization and tissue integrity of the NEC model over time. Based on TEER measurements, NEC cultures achieved peak epithelial barrier integrity about 7 days after ALI before decreasing through day 21. TEER values increased in all cell cultures following ALI (Figure 1L).

To confirm NEC multilayers differentiated biologically we evaluated mucin production by Western dot blot analysis of 20 ug of protein from both ALI and LLI cultures. Six targets were analyzed, including respiratory tract associated mucins MUC1, MUC2, MUC4, MUC5AC, and MUC5B (Singh and Hollingsworth, 2006) and Mel-CAM as a negative control. ALI but not LLI protein lysates showed strong production of MUC1 (data not shown). MUC2, MUC5AC, and MUC5B were minimally detected in ALI lysates but again were not observed in the LLI cultures. MUC4 and Mel-CAM (negative control) were not detected in either culture condition.

Finally, Illumina RNA-Seq performed on poly(A) enriched RNA characterized transcriptional changes in NEC after immortalization with HPV E6/E7. Comparing gene expression between primary cells (reference) and immortalized (experimental) yielded 36,536 genes with readable counts. Utilizing the Wald test, we found 2,146 genes significantly (*p* < 0.05) changed in immortalized cells. Of the 2,146 genes, 1,497 were found to have a baseline mean read count of at least 100. Of these 1,497 genes, 235 were noted to have at least a 5-fold change in expression. We removed unannotated genes from this final set, yielding 228 genes, 47 of which were found to be downregulated and 181 upregulated in immortalized cells. All downregulated genes were imported into the STRING database using cluster-specific gene names, yielding no significantly enriched pathways. Genes that showed upregulated expression in immortalized cells were imported into the STRING database and a total of 4 enriched pathways were found within the KEGG analysis (Supplementary Figure 1). Consistent with immortalization outcomes, KEGG pathways for cell cycle, cell senescence, p53 cell signaling and DNA replication were upregulated. Importantly, no pathways related to inflammation, immune response or other mucosal signaling cascades were impacted supporting the conclusion that the immortalized cells will create similar microenvironments to the primary cultures.

### NEC Multilayers Supported Colonization by Transplanted Nasal Microbiomes and MRSA

**Figure 2:**
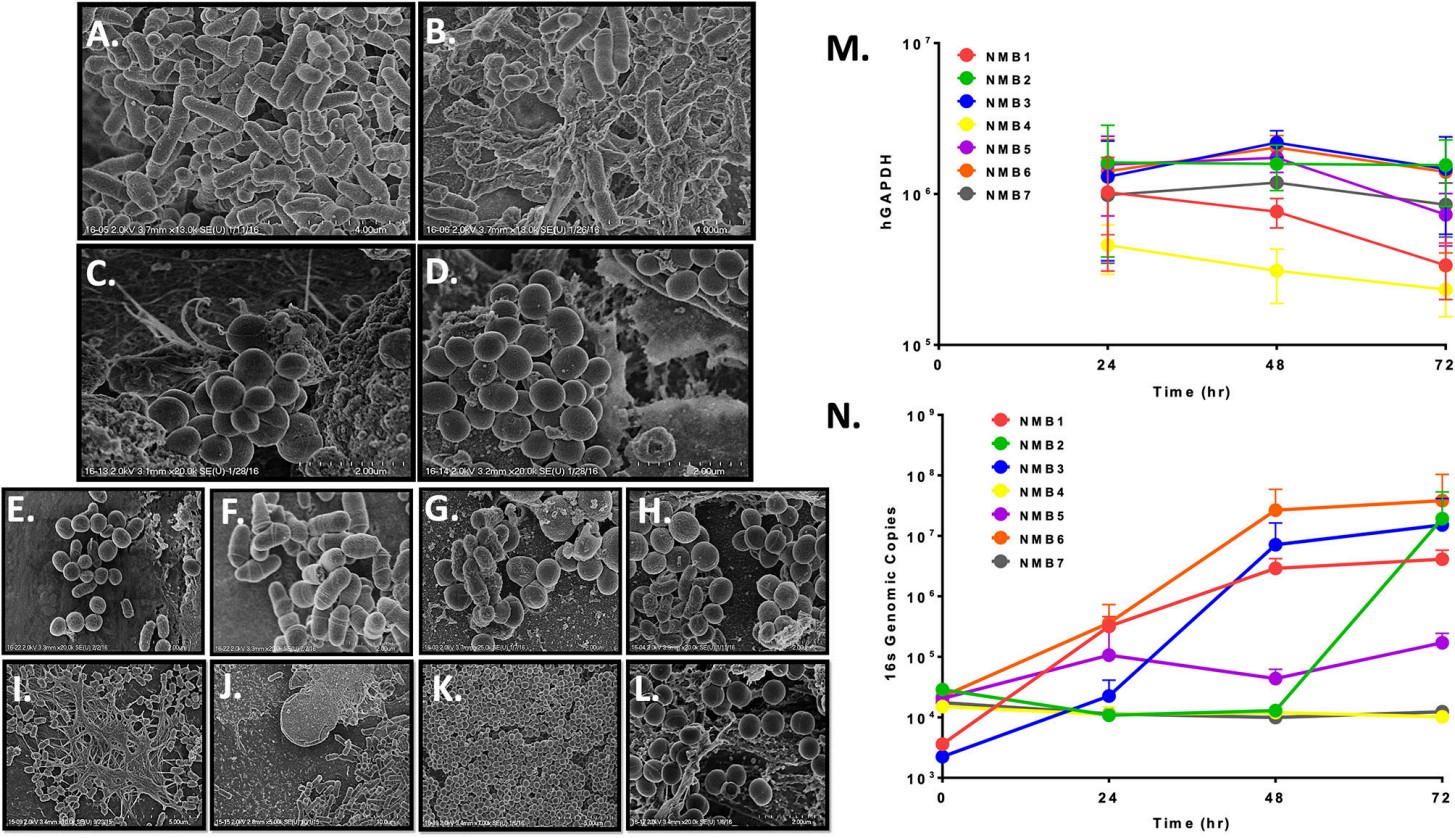
Representative NEC multilayers supported colonization of microbial communities as revealed by SEM imaging at 5–20 k magnification **(A–L)** and qPCR **(M,N)**. **A–B**. NEC03 multilayers seeded with NMB from a healthy donor revealed numerous bacterial cell morphologies, predominantly rods and cocci. **C–D**. NEC03 multilayers seeded with NMB from a donor who recently received antibiotic therapy revealed predominantly clustered cocci, consistent with *Staphyloccocus spp*. **(E–J)** NEC03 multilayers seeded with NMB were examined at 4 **(E,F)**, 24 **(G,H)** and 48 h **(I–J)**. Proliferation and stabilization of bacterial communities were most evident by 24 and 48 h, with extracellular polymeric substances deposition observed at 48 h **(I)**. **(K,L)**. NEC02 multilayers seeded with *S. aureus* displayed confluent colonization by the bacteria. **(M)** Health of the NEC02 was maintained in the presence of 7 distinct NMB over 72 h as indicated by genomic counts (hGAPDH). **(N)** NEC02 multilayers supported bacterial colonization by distinct NMB over time.

The goal of the NEC multilayer culture system was creation of an *ex vivo* environment that would support reproducible colonization by relevant single species and nasal microbiota communities. To test this aspect of the immortalized NEC multilayers, we collected nasal microbiomes (NMB) from healthy donors and after molecular characterization of the bacterial communities, we diluted cryopreserved aliquots to a concentration of 1 × 10^5^ genome equivalents per 10 ul inoculum that was applied to the mature apical surface of multilayers created in 96 well transwells similar to our previous approach in vaginal multilayer cultures (Rose et al., 2012; Pyles et al., 2014). This concentration preserved minor species and allowed for bacterial replication as part of the culture colonization process. In an initial study, NMB communities collected from sisters who lived together were used to colonize matured NEC because one had recently completed a course of antibiotics. Molecular characterization of the samples confirmed distinct profiles indicating 12 bacterial species were present in the untreated sister (Sister 1) with nearly equivalent proportions of Moraxella and *Staphylococcus epidermidis* (Supplementary Figure 2). The community in the second sister following her antibiotic course was dominated by *S. epidermidis* and contained only 8 detected organisms (Supplementary Figure 2). The sisters' NMBs, seeded onto NEC culture triplicates, were observed by SEM and revealed the establishment of 2 visually distinct microbial communities (Figures 2A–D). The NMB from the sister who had not taken antibiotics (Sister 1) was morphologically diverse with abundant bacteria of many morphotypes (Figures 2A,B). In contrast, the post-antibiotic NMB (Sister 2) produced a strikingly homogenous colonization by clusters of cocci consistent with *Staphylococcus spp*. (Figures 2C,D). These bacterial communities served as proof of concept that the cultured environment was capable of supporting nasal microbiota colonization.

SEM imaging of matured NEC fixed 4, 24, and 48 h post inoculation confirmed bacterial attachment to NEC structures at 4 h followed by proliferation and stabilization of diverse bacterial communities at 24 and 48 h (Figure 2E–J). Imaging of selected NMBs in matured NEC illustrated flourishing communities of spatially separated but morphologically heterogeneous groups of bacteria as well as fields illustrating complex landscapes similar to *in situ* bacterial colonization of nasal mucosa with organisms occupying various topographical mucosal niches (Hibbing et al., 2010; Yan et al., 2013; Proctor and Relman, 2017). Finally, extracellular polymeric substances consistent with biofilm formation were evident in some NMBs at both 24 and 48 h (Figure 2I). Importantly, micrographs of the cultured NMBs depicted colonized bacteria limited to the apical aspect of NEC and provided no evidence of bacterial invasion of the cultured tissue and no indications of negative impacts on the NEC multilayer.

The appearance of NEC cultures colonized with a monoculture inoculum of MRSA also was compared to NMBs that contained *S. aureus* as a community member. While cultures of *S. aureus* seeded within a heterogeneous community, as shown by the previously cultured NMBs (Figure 2E–J), established spatially separated patches, NEC cultures seeded with only *S. aureus* revealed widespread and confluent colonization (Figure 2K) consistent with unrestrained growth. Despite the MRSA proliferation, the colonization still appeared to remain limited to the apical surface with no visible mucosal invasion (Figure 2L).

To more accurately quantify bacterial expansion, replication kinetics of NMBs transplanted to matured NEC multilayers were further investigated through PCR quantitation of the bacterial 16S gene at 0, 24, 48, and 72 h post inoculation. Importantly, the general health of the NEC cultures at 48h after colonization was maintained based on quantification of human glyceraldehyde-3 phosphate dehydrogenase (hGAPDH) DNA as a marker of NEC genomes (Figure 2M) and by TEER measurements (data not shown). Following inoculation with NMBs diluted to as few as 10^2^ to 10^3^ genome equivalents, exponential bacterial proliferation was observed until 48 h, when genomic counts appeared to stabilize or decline (Figure 2N) consistent with a microbial steady state rather than unrestrained bacterial replication. The results indicated that at 72 h post NMB inoculation, the bacterial cultures of NMBs 1–7 did not significantly decrease hGAPDH levels compared to sterile controls. Interestingly, quantified hGAPDH was significantly elevated in cultures inoculated with NMBs 2 and 3 compared to the sterile control (Figure 2M).

### NEC Multilayers Supported Reproducible Colonization of NMB Communities

**Figure 3:**
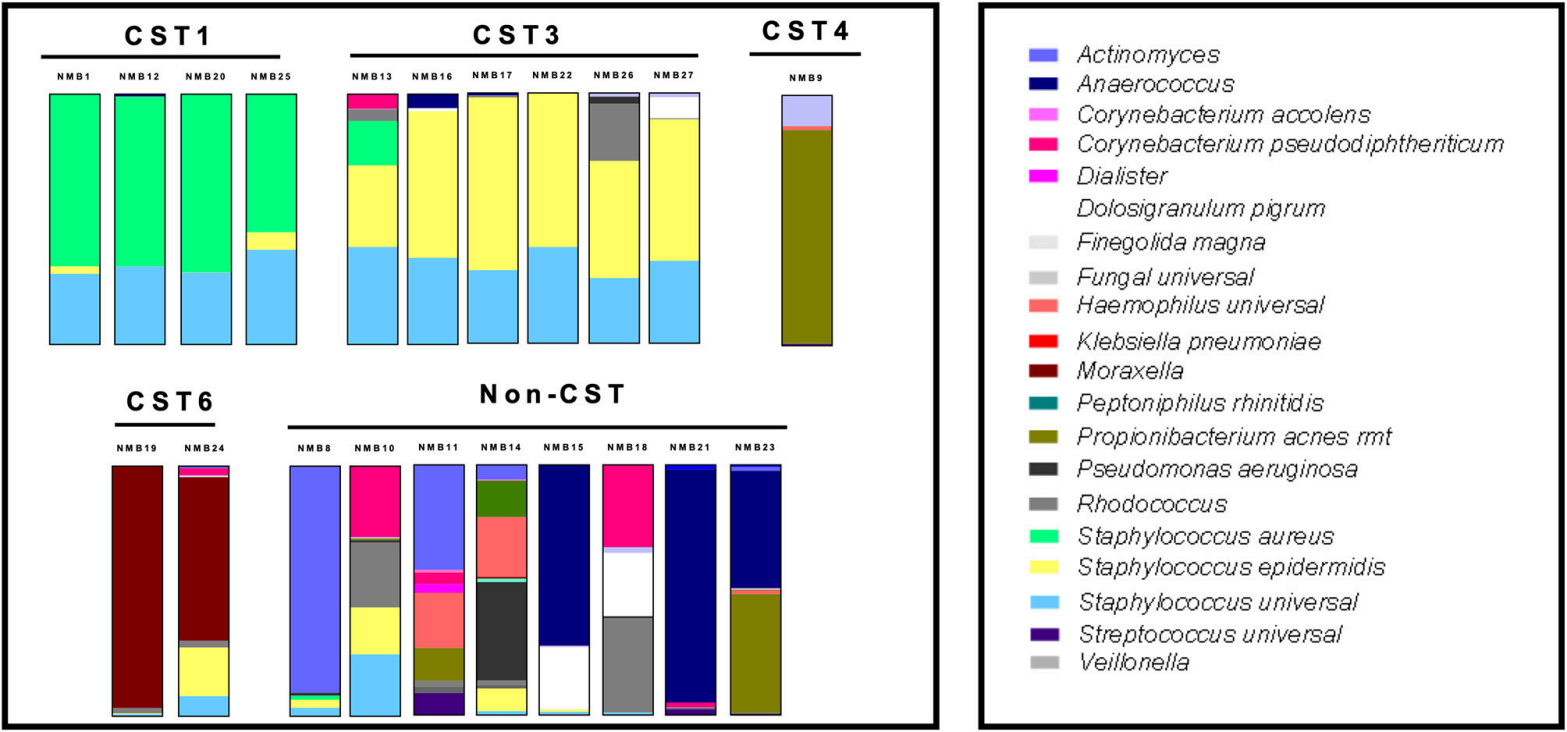
Diverse profiles of transplanted human NMB communities after 48 h of cultivation on mature NEC ALI cultures. CST1, 3, 4, and 6 community profiles were reproducibly cultivated in concordance with the donor profiles. Eight communities that did not match a CST also were successfully cultivated. These communities were tested extensively on NEC03, 05, 08, and 09 with average proportional bar charts presented (*n* = 3–6 replicates for each of the 4 NEC).

**Figure 4:**
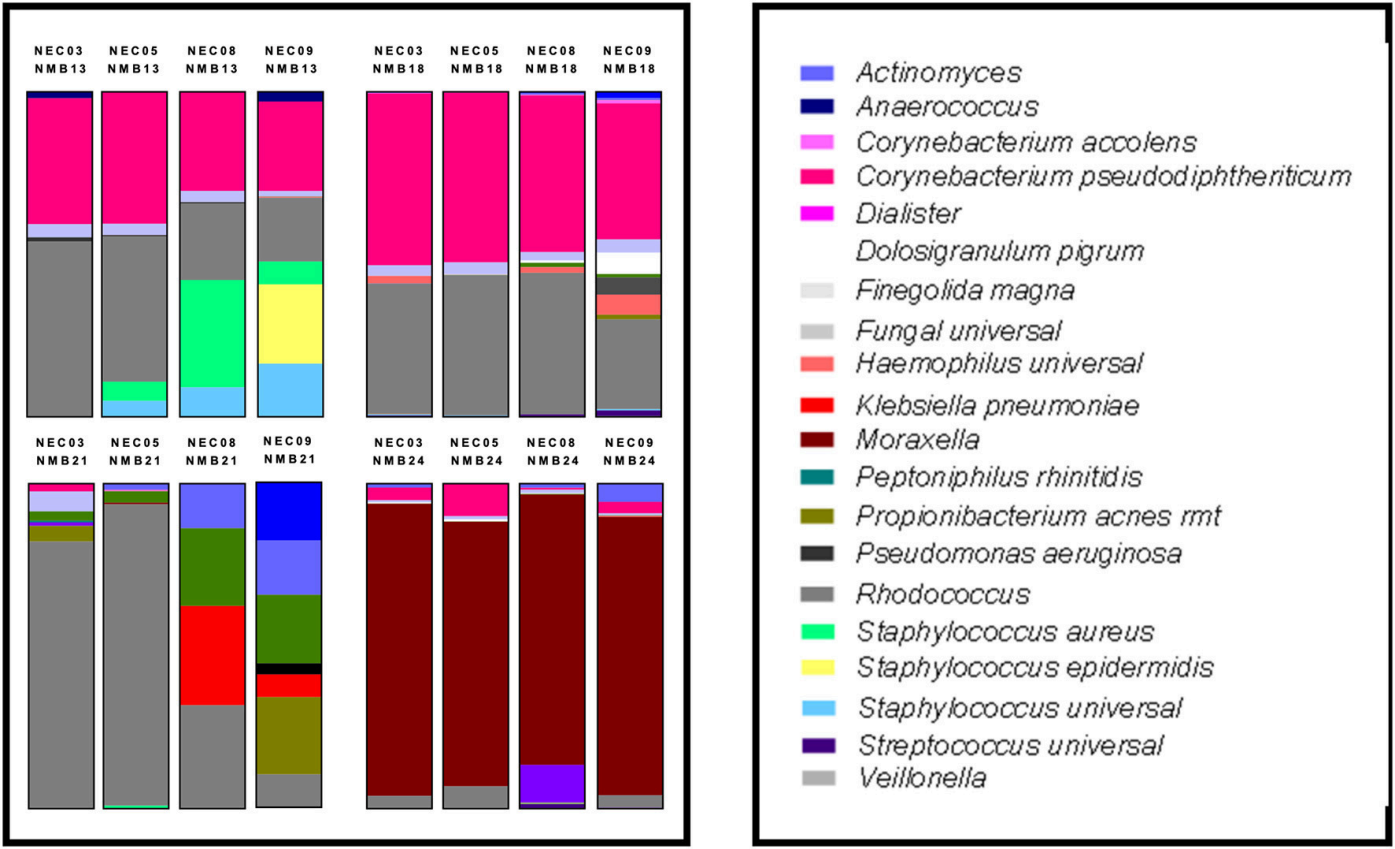
Human NMB colonization of 4 NEC ALI cultures. Selected NMB inocula were transplanted onto the apical surfaces of 4 distinct NEC cultures to examine differences in colonization capacity. For these studies a single cryopreserved aliquot of NMB was prepared and used to inoculate each of the NEC in parallel. These analyses revealed that the NEC09 microenvironment led to the greatest variance in community profile relative to the other cultures. Each profile represents the average of triplicate wells created in parallel. The study was repeated with indistinguishable results.

To molecularly profile the transplanted NMB communities, we employed a customized qPCR array (See Supplementary Table 1) that evaluated 46 bacterial targets as well as total bacteria (universal 16S) and host genomes (hGAPDH). Data from these assays were plotted in standard relative abundance proportional bar charts to illustrate diversity of *in vivo* commensal bacterial communities from the collected nasal swabs (Figure 3). These samples were selected to expand the number of tested CSTs and included 20 communities from new donors and NMB1 that was used in the previous studies. Unfortunately, local samples failed to identify representative communities for each of the 7 CSTs, but did provide multiple examples for CST1, 3 and 6 as well as a single CST4 and a group of communities that did not easily fit into a CST (Liu et al., 2015). Following dilution and transplant to a cultured human mucosa distinct from the donor, polymicrobial NMB communities were consistently and reproducibly established with similar overall profiles with noted exceptions (Figure 4). From our repository of human NMB samples, our NEC model supported reproducible cultivation of the 4 distinct CSTs and a number of non-CST profiles.

To compare the impact of microenvironments created by NEC derived from male and female tissues of disparate genetic backgrounds, we selected 2 male (NEC03 and 05) and 2 female cultures (NEC08 and 09). Mature ALI cultures for each of the 4 donors were created in parallel and were then colonized as triplicates by single stocks of 4 selected NMBs representing communities from male and female donors (NMB13, NMB18 were from female donors while NMB21, NMB24 were from males). DNA was analyzed from each of three wells per condition at 48 h after the cultivated community stabilized and analyzed, first with customized qPCR array, and then with single target qPCR assays for selected dominant organisms among the communities (Figure 4). These targets included *C. pseudodiphtheriticum, Enterococcus spp., Klebsiella spp, Moraxella spp., Rhodococcus spp., S. aureus* and *S. epidermidis*. Despite both gender and genetic distinctions in the 4 NEC cultures, no significant differences in absolute abundance were observed for any of the cultivated NMBs (p>0.05; data not shown) with two exceptions. The original NMB13 had a small proportion of *S. epidermidis* that was evident in the communities cultivated in the NEC09 wells but not in the other three cultures. Secondly, the *Moraxella spp* levels in NEC03 inoculated with NMB24 (average genomic copy of 6.04log10 ± 5.88 SDEV) were significantly higher than those in the other three cultures (*p* < 0.01, ANOVA).

## Discussion

We present results outlining the development of a model system that employs immortalized NEC cultured in conditions leading to differentiation and creation of human nasal mucosa multilayer structures. These ALI cultures support reproducible colonization by communities of diverse commensal nasal bacteria transplanted from swabs collected from asymptomatic donors. The immortalized nature of the NEC from several haplotypes of both genders eliminates the limitations associated with primary cell senescence and allows for repeated culture studies including colonization by NMB communities or single species of relevant bacteria. Methodological outcomes for molecular analysis of host-microbe and microbe-microbe interactions included harvested DNA, RNA and proteins from both the apical and basolateral chambers modeling the nasal lumen and the systemic compartments of the host, respectively. These distinct samples may be further utilized for screening of the impact of distinct communities associated with potential clinical outcomes to inform subsequent clinical research studies.

The model development established optimal NEC seeding density in both 24 and 96 well transwell formats as indicated by thorough evaluation of the maturation point and longevity of matured multilayers. This initial work also confirmed the value of the ALI culture format over standard liquid interface. As reported previously, formation of tight junctions correlated with TEER measurements (Ehrhardt et al., 2002; Grainger et al., 2006; Papazian et al., 2016) that identified the optimal maturation time to be roughly 7 day after establishing the air interface for NEC from each of the 12 donors. Further, the 7 day TEER values of the immortalized NEC cultures were comparable to those of ALI primary NEC cultures (300–800 Ωcm^2^) and greater than measurements typical of the carcinoma derived nasal cell line RPMI2650 (80–200 Ωcm^2^) (Lopez-Souza et al., 2003, 2009; Lin et al., 2007; Wengst and Reichl, 2010; Thavagnanam et al., 2014; Ong et al., 2016; Papazian et al., 2016). However, other transformed nasal and respiratory cell lines, including RPMI2650 and 16HBE14o-, produce multilayers 10–15 cell thick and lacked necessary differentiation outcomes (Ehrhardt et al., 2002; Wengst and Reichl, 2010; Kreft et al., 2015). Unlike standard monolayer cultures, matured ALI NEC multilayers also produced mucins consistent with nasal secretions and, perhaps most importantly, created the necessary microenvironment to support colonization by both relevant single bacterial species and polymicrobial communities.

Evaluation of the relative abundance of bacteria in cultured communities revealed that the environments created by the NEC cultures preferentially supported some community members. Specifically, *Staphylococcus spp*. were consistently increased in relative abundance compared to the inocula profile. In such cultivated communities the overall bacterial load was not significantly higher than other cultivated community levels (p>0.05) suggesting that these were simple alterations of the proportions of the bacteria in the community and, as noted above, did not reflect unrestrained growth. In addition, throughout the optimization process we determined our culture system to be highly amenable to colonization with *S. epidermidis* (Figure 4, NMB13 NEC09), both in communities known to harbor the organism and as an environmental contaminant. This organism became a dominant community member but also did not overgrow. In contrast, most communities that included even minor proportions of *Pseudomonas aeroginosa* showed increases in overall 16S DNA levels and nearly complete dominance by this organism consistent with unrestrained bacterial expansion (e.g., Figure 3, NMB23) suggesting that some *Pseudomonas spp*. are not compatible with this culture system. The differences observed in community profiles established in NEC09 suggested a different microenvironment that may reflect a different differentiation state or modeled tissue outcome for this culture relative to the other three. Additional molecular studies will be required to determine if these cells are distinct from the other cultures based on host genetics and/or the represented tissue.

Likened to a chemostat, the human body constantly generates nutrients that sustain bacterial colonies and, based on the nutrient-niche hypothesis, competition for limited nutrients pressures for microbial resource partitioning (Brown et al., 2008; Krismer et al., 2014). Viewing microbial ecosystems through this lens implies that members of bacterial communities phenotypically differentiate to assume *in situ* niches metabolizing different energy sources, and, in accordance with the *in situ* microenvironment, this contributes to controlled bacterial colonization (Brown et al., 2008; Consortium, 2012). Clinical studies however are confounded by the behavior, diet and a myriad of other factors that ultimately change the status of the mucosal surfaces in subjects thereby altering the available nutrients utilized by the bacteria. On average, molecular analysis of clinical nasal secretions reveals limited amounts of glucose and amino acids, suggesting that in addition to competing for simple nutrients, nasal commensals may take advantage of novel metabolic pathways in response to *in situ* stimuli (Krismer et al., 2014). The presented culture system provides opportunities to control many of the factors leading to changes in the microenvironment as well as colonizing the same modeled host mucosa with distinct NMBs. As additional support of the utility of the model, NMB transplants established steady state populations as indicated by SEM and 16S loads over time without any obvious damage to the multilayer. This controlled colonization supported the integrity of the model's epithelial barrier and negated the concerns that sustained exponential growth would be supported by the artificial culture environment. In contrast, colonization by the nasal mucosal pathogen MRSA led to uniform, high titer colonization after just 18 h leading to the conclusion that this organism found an optimal and unrestricted environment as a monoculture when compared to the heterogeneous NMBs. However, when MRSA was part of a community we observed its growth to be restrained. *Pseudomonas spp* also showed high titer propagation in the NEC culture system leading to the highest bacterial levels observed regardless of the composition of the bacterial communities tested to date. These outcomes are limitations of the model system but present opportunities for understanding the components of the microenvironment that support colonization by these pathobionts.

In humans, nasal bacteria colonize the turbinates in spatially separated communities capable of altering neighboring communities through a variety of mechanisms (Hibbing et al., 2010; Yan et al., 2013). Elucidation of interspecies bacterial interactions has demonstrated clinical relevance including a variety of indications of exclusion or increased susceptibility to colonization by pathogens. As an example, after correlating elevated *Corynebacterium spp*. colonization in pediatric patients with disproportionately low *Streptococcus pneumoniae* colonization, Bomar et al. found that metabolic activity of colonized *C. accolens* inhibits growth of *S. pneumoniae* through hydrolysis of human skin surface triglycerides into free fatty acids (Bomar et al., 2016). Another case of interspecific competition is the synthesis and secretion of the peptide Lugdunin by nasally residing *Staphylococcus lugdunensis* that demonstrated significant antimicrobial activity against *S. aureus* (Zipperer et al., 2016). The impact of a bacterial interspecies interaction is not limited to direct competition with other examples, including secretion of antimicrobial compounds and intercellular cooperative behaviors (Hibbing et al., 2010; Wos-Oxley et al., 2010; Yan et al., 2013; Proctor and Relman, 2017). Certain commensal organisms sharing relative abundance and distribution across clinical samples even suggest evidence of interspecies bacterial community symbiosis (Wos-Oxley et al., 2010). Recent NGS evaluation of clinical samples indicates that the abundance of *D. pigrum* indirectly correlates with the likelihood of *S. aureus* presence, suggesting interspecies bacterial exclusion of the pathogen *S. aureus* may be of value as a clinical therapy (Liu et al., 2015). Future studies using the established NEC model to test the impact of modification of NMBs on subsequent challenge with pathogens should help to predict what constitutes a “healthy” microbiota based on clinical associations as described above.

Immortalization of human epithelial cells with HPV16 E6/7 creates an intermediate between primary cell and carcinoma-derived human cell cultures facilitating easily replenished materials of a constant genetic background through the enhanced lifespan of serially passaged heterogeneic cultures that preserve a greater capacity of cellular differentiation than neoplastic cell lines (Chun et al., 2002; Gudjonsson et al., 2004; Rose et al., 2012). Although these cells are able to be passaged *ad infinitum*, extended growth will allow for transformation associated with typical cancer progression. In fact, the E6/E7 immortalization can cause subsequent mutations, including duplication of chromosomes 5 and 20 and losses on chromosome 9 (Tsao et al., 2002; Zabner et al., 2003). To limit this concern, we therefore passage the cultures no more than 40 times before thawing a fresh stock. An additional limitation of the current culture configuration is the absence of other human cell types beyond NEC. In preliminary studies we have successfully introduced macrophage and plan future studies to examine the effects of dendritic cells and macrophage on culture phenotypes in the presence of selected NMB communities.

This system also relies upon cryopreserved aliquots of NMB samples that can be reliably used over time to reproducibly colonize NEC cultures. Using methods optimized for vaginal microbiome community preservation (Rose et al., 2012), we optimized preservation of both pediatric and adult communities for these studies. With our optimized freezing method, we established that the number of viable bacteria was not significantly altered, however occasional aliquots failed to produce similar communities when used to colonize NEC cultures. In future studies we intend to establish if length of cryopreservation affects potential viability and/or profiles of the NMBs. A noted limitation of the model system involves the dilution of the bacterial communities to allow for colonization and stabilization leading to some low titer organisms that are lost or only observed sporadically. This has been observed in a number of studies as shown in Figure 4 NMB13 for *S. epidermidis*. A second limitation involved the observation that, across NMBs, specific bacteria found a favorable microenvironment leading to increased relative abundance or in some cases, including some *P. aeruginosa* strains, unrestrained growth that compromised the NEC cultures. Specifically, *Staphylococcus spp*. were often favored by the modeled microenvironment as evidenced by increased relative abundance in the cultured bacteria community composition. While these bacteria were favored by culture conditions, they were still successfully co-cultured with other NMB bacteria creating novel community profiles. Finally, introduction of fungal species contained in NMB communities also resulted in rapid overgrowth and destruction of the NEC culture.

Together, the data supports the conclusion that this model offers expanded utility and reproducibility for testing of host-microbe and microbe-microbe interactions relating to nasal mucosa. The establishment of NEC cultures from multiple donors of both gender and several haplotypes adds to the experimental opportunities. The air interface better models the nasal lumen for testing of inhaled therapies and probiotics in the context of a colonized mucosal surface and may be useful for preclinical screening of test articles prior to clinical trials.

## Ethics Statement

Collection of NMB from healthy volunteers was approved by the institutional review board at University of Texas Medical Branch. Adults, 18 years and older filled out a brief questionnaire and provided written consent before being assigned a unique study number. Each participant was instructed on sample collection and observed during the nasal swabbing. De-identified, discarded tissue was collected from sinonasal procedures performed by the University of Texas Medical Branch Otolaryngology Department or from nasal tissues collected from willed bodies through an approved research protocol with the National Disease Research Interchange (NDRI; Philadelphia, PA, USA).

## Author Contributions

RP, TE-P, MC, and VP study design and conception. DC, JF, SH, CM, MC, AM, AM-C, YS, and SW performance of experiments. DC, JF, RP, AM, and SW data analysis. DC, JF, SH, AM, SW, and RP manuscript production. All authors approved of this manuscript.

### Conflict of Interest Statement

The authors declare that the research was conducted in the absence of any commercial or financial relationships that could be construed as a potential conflict of interest.

## Supplementary Material

Supplementary Figure 1 Significantly upregulated genes identified by RNA-seq in male and female immortalized NEC were identified by comparison to data from equivalent primary cultures created in parallel. The gene id's were loaded into the STRING database and KEGG analysis was performed. The 4 significantly enriched pathways identified were expected and clearly related to immortalization: Cell Cycle, Cellular Senescence, p53 pathway, and DNA replication. No other pathways were identified as significantly upregulated compared to primary cultures processed in parallel.

Supplementary Figure 2 Proportional bar charts of the two sisters' NMBs. The community from Sister 1 was the non-antibiotic control and the sample from Sister 2 was post-antibiotic. We found 12 bacterial species in the NMB of Sister 1, whereas only 8 were found in Sister 2. The community from Sister 2 produced an overgrowth of *S. epidermidis* as determined by our NMB array.

Supplementary Table 1 Primers, references and details of the custom NMB qPCR array. The thermocycling protocol was previously described (Veselenak et al., 2015) with slight modification as follows: 8 cycle amplification of 95°C for 30 s, 48°C for 30 s, and 72°C for 30 s followed by a 40 cycle amplification of 95°C for 15 s, 56°C for 20 s and 72°C for 20 s during which real time data were acquired at the annealing step. Bio-Rad CFX Manager software was used to complete Cq analysis with a constant baseline adjustment of 50 relative fluorescent units for all array runs. Melt temperatures (Tm) for each amplicon were compared to historical values with an acceptable range of ±0.8°C prior to data analysis. A list of the primers and cycling program employed for the customized NMB qPCR array.
