# Supplementary figures and images for: Development of a Novel ex vivo Nasal Epithelial Cell Model Supporting Colonization With Human Nasal Microbiota
Source: Front Cell Infect Microbiol. 2019 May 21;9:165. doi: 10.3389/fcimb.2019.00165 (PMC6536665; doi:10.3389/fcimb.2019.00165)

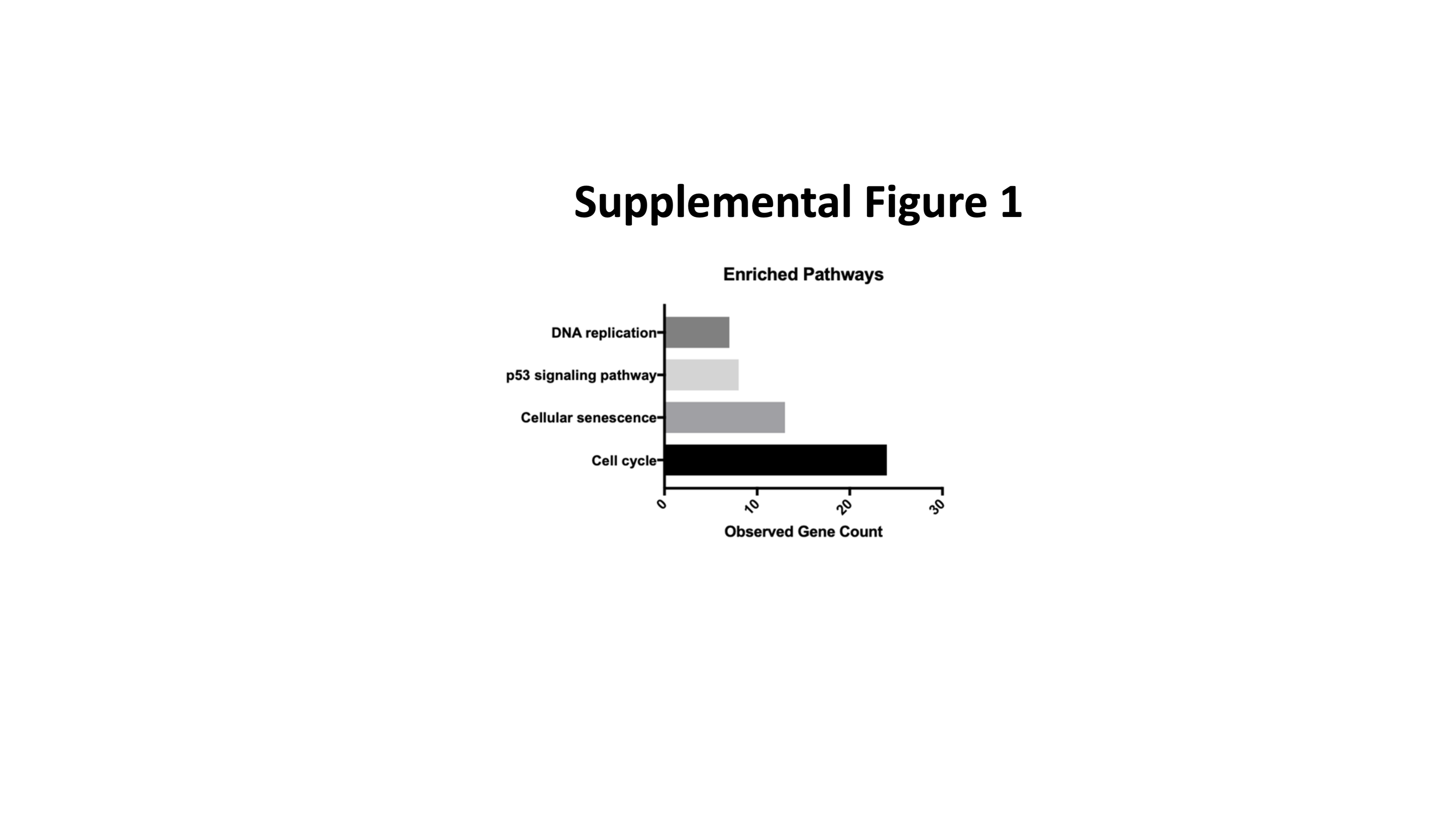

Supplement: Supplementary Figure 1 — Significantly upregulated genes identified by RNA-seq in male and female immortalized NEC were identified by comparison to data from equivalent primary cultures created in parallel. The gene id's were loaded into the STRING database and KEGG analysis was performed. The 4 significantly enriched pathways identified were expected and clearly related to immortalization: Cell Cycle, Cellular Senescence, p53 pathway, and DNA replication. No other pathways were identified as significantly upregulated compared to primary cultures processed in parallel. [file Image_1.TIFF]

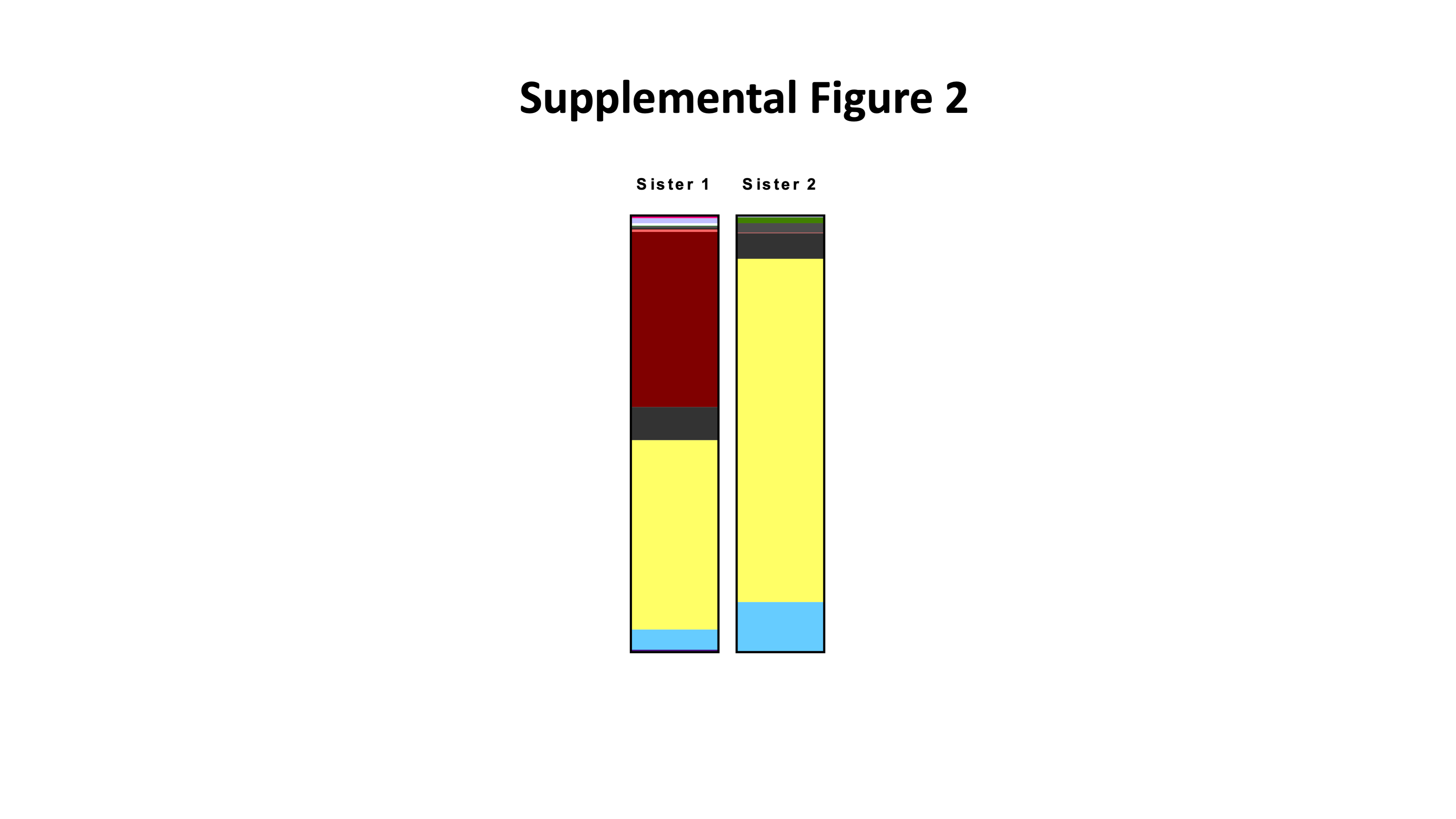

Supplement: Supplementary Figure 2 — Proportional bar charts of the two sisters' NMBs. The community from Sister 1 was the non-antibiotic control and the sample from Sister 2 was post-antibiotic. We found 12 bacterial species in the NMB of Sister 1, whereas only 8 were found in Sister 2. The community from Sister 2 produced an overgrowth of S. epidermidis as determined by our NMB array. [file Image_2.TIFF]
