# Supplementary material for: Development of a Novel ex vivo Nasal Epithelial Cell Model Supporting Colonization With Human Nasal Microbiota
Source: Front Cell Infect Microbiol. 2019 May 21;9:165. doi: 10.3389/fcimb.2019.00165 (PMC6536665; doi:10.3389/fcimb.2019.00165)
Supplement: Supplementary Table 1 — Primers, references and details of the custom NMB qPCR array. The thermocycling protocol was previously described (Veselenak et al., 2015) with slight modification as follows: 8 cycle amplification of 95°C for 30 s, 48°C for 30 s, and 72°C for 30 s followed by a 40 cycle amplification of 95°C for 15 s, 56°C for 20 s and 72°C for 20 s during which real time data were acquired at the annealing step. Bio-Rad CFX Manager software was used to complete Cq analysis with a constant baseline adjustment of 50 relative fluorescent units for all array runs. Melt temperatures (Tm) for each amplicon were compared to historical values with an acceptable range of ±0.8°C prior to data analysis. A list of the primers and cycling program employed for the customized NMB qPCR array. [file Table_1.DOCX]

| **NMB Target** | **Forward Primer** | **Reverse Primer** | **Reference** | **PMID** |
| --- | --- | --- | --- | --- |
| *Acidovorax spp* | GCGAAAGCTTTGCTAATAC | GGCTTGGTAAGCTTTTATC | This study |  |
| *Acinetobacter spp* | CGTTCAACCGTGTAAAATTACGTAA | ACCACCAGAAGTATCACCACG | Journal of Clinical Microbiology 2006:42; 2921-2932 | 16891513 |
| *A. calcoaceticus* | ACTGTTCCTGCTCGTAAG | TGGTCAATAATGGATAATCTTCTG | This study |  |
| *Actinomyces spp* | CTGGCTTGAGTGCAGTAG | CGGTGTTCCTCCTGATAT | This study |  |
| *Aeromonas spp* | GCATTTGAAACTGACAAG | TATTCCTCCAGATCTCTAC | This study |  |
| *Alloiococcus spp* | AAAAGAATTGACGGGGAC | CAAGAGCTGGTAAGGTTC | This study |  |
| *Anaerococcus spp* | TGGGGAATTTTGCACAAT | GTATGGTACTGTCATTTTCTTC | This study |  |
| *Cloacibacterium spp* | CGGCATCGTTTAATATTGAA | CCCCTAAAAGATCATCGC | This study |  |
| *Corynebacterium spp* | GGGTAATGGCCTACCAAG | CCGTATCTCAGTCCCAATG | This study |  |
| *C. accolens* | CGGATAACAATCTCAATGC | GTCAACCTCGTACTCCTC | This study |  |
| *C. pseudodiphtheriticum* | AGCAACATCGCCATCCATAA | CGCCACGAGAAATTGATTAAAGAA | This study |  |
| *Dialister spp* | CCTAGTGTAGCGGTGAAA | GTCAGTTTTCGTCCAGAA | This study |  |
| *Dolosigranulum pigrum* | TATCTAAGTGCTCAGTCA | GCGTGAACTATAATGTAGA | This study |  |
| *Escherichia coli* | GTGTGATATCTACCCGCTTCGC | AGAACGGTTTGTGGTTAATCAGGA | Journal of Microbiological Methods 52 (2003) 123– 131 | 12401234 |
| *Enterococcus spp* | AGAAATTCCAAACGAACTTG | CAGTGCTCTACCTCCATCATT | Water Research 39 (2005) 559–568 | 15707628 |
| *Finegoldia magna* | ATTGGATGGTTACAGAGTT | CCAGTAATAATGTTAAGAGTGAT | This study |  |
| *Fusobacterium spp* | TGGTTATRTAAGTCTGATGT | CACTTGTAGTTCCGCTTA | This study |  |
| *Haemophilus spp* | GGAGTGGGTTGTACCAGAAGTAGAT | AGGAGGTGATCCAACCGCA | Journal of Clinical Microbiology, May 1998, p. 1185–1188 | 9574673 |
| *H. influenzae* | ATGGCGGGAACATCAATGA | ACGCATAGGAGGGAAATGGTT | Clin Microbiol Infect 2015:21;788e1-788e13 | 25980353 |
| *Klebsiella pneumoniae* | AGGCCGAATATGACGAAT | GGTGATCTGCTCATGAA | Clin Microbiol Infect 2015:21;788e1-788e13 | 25980353 |
| *Lachnospiraceae spp* | ATCCGGATTTACTGGGTG | CTCCAAATATCTACGCATTTC | This study |  |
| *Lactobacillus spp* | CTCAAAACTAAACAAAGTTTC | CTTGTACACACCGCCCGTCA | FEMS Microbiology Letters 214 (2002) 271-275 | 12351242 |
| *Moraxella spp* | CTTCCTGGCATCATACTG | CAACGACTGGTAGACATC | This study |  |
| *M. catharrlis* | GAGCGTGAATGTATTATG | ATCTGTCATCAATGGAAT | This study |  |
| *Neisseria spp* | GAAACCAAAGTCAAAGTCATC | CTGATACCTTCGCCGTAA | This study |  |
| *Peptoniphilus spp* | GACCGGTATAGAGATATACCCT | CACCTTCCTCCGATTTATCATC | J Clin Microbiol 2007: 45; 3270-3276 | 17687006 |
| *P. rhinitidis* | TTCAACAATTCCTTCACTAACTT | ATACGAACATGGCGATAGAT | This study |  |
| *Peptostretococcus spp* | TCATAGGAGGAAGCCCTGGCTAAA | TAAGCTCCACGCTTTGACACCTGA | J Clin Microbiol 2010: 48; 1812-1819 | 20305015 |
| *Prevotella spp* | GGGATGCGTCTGATTAGCTTGTT | CTGCACGCTACTTGGCTGGTTC | J Clin Microbiol 2010: 48; 1812-1819 | 20305015 |
| *Propionibacterium acnes* | TTGCCAACAAATTGACTTTA | GGCTGTTCTTGGTAGAAG | This study |  |
| *Pseudomonas spp* | ACTTTAAGTTGGGAGGAAGGG | ACACAGGAAATTCCACCACCC | FEMS Microbiol Lett 333 (2012) 77–84 | 22639954 |
| *P. aeruginosa* | CCTGACCATCCGTCGCCACAAC | CGCAGCAGGATGCCGACGCC | Clin Microbiol Infect 2015:21;788e1-788e13 | 25980353 |
| *Rhodococcus spp* | TACGGGCAGACTTGAGTA | GGTGTTCCTCCTGATATCT | This study |  |
| *Rothia spp* | GCWAACTAGAGTGCAGTAG | CGGTGTTCYTCCTGATAT | This study |  |
| *Ruminococcaceae* | CAACGCGAAGAACCTTAC | ACGGCAGTCCTATTAGAG | This study |  |
| *Sphingomonas spp* | GAGACTGGATTGCTTGAA | GTTCTTCCGAATATCTACGA | This study |  |
| *Staphylococcus spp* | GAACGTGGTCAAATCAAAGTTGGTGAAGA | GTCACCAGCTTCAGCGTAGTCTAATAA | J Clin Microbiol, Oct. 2009, p. 3129–3137 | 19710268 |
| *S. aureus* | ACAAAGGTCAACCAATGACATTCAGACTA | AAATGCACTTGCTTCAGGGCCATAT | J Clin Microbiol, Oct. 2009, p. 3129–3137 | 19710268 |
| *S. epidermidis* | GTAGCAATACAGACACTA | CCACCAATATAGACAACT | This study |  |
| *Streptococcus spp* | AGTCGGTGAGGTAACCGTAAG | AGGAGGTGATCCAACCGCA | Journal of Clinical Microbiology, May 1998, p. 1185–1188 | 9574673 |
| *S. pneumoniae* | ACGCAATCTAGCAGATGAAGCA | TCGTGCGTTTTAATTCCAGCT | Clin Microbiol Infect 2015:21;788e1-788e13 | 25980353 |
| *Veillonella spp* | GACGAAAGTCTGACGGAG | CCGATTAACAGAGCTTTACAA | This study |  |
| Adenovirus | GCCACGGTGGGGTTTCTAAACTT | GCCCCAGTGGTCTTACATGCACATC | Journal of Clinical Virology 41 (2008) 53-56. | 18093871 |
| Bocavirus | TGCAGACAACGCYTAGTTGTTT | CTGTCCCGCCCAAGATACA | [J Clin Microbiol. 2006 Sep;44(9):3231-5.](javascript:AL_get(this,%20'jour',%20'J%20Clin%20Microbiol.');) | 16954253 |
| CMV | GGACGCCGAACTCATGGA | AGGTGGCTTGACGTATTTGAGAA | J Virol 2004 78; 10360-103610 | 15367602 |
| Fungus | TCCTCCGCTTATTGATATGC | GCATCGATGAAGAACGCAGC | PCR Protocols: 1990: White et al;315-322 |  |
| 16s | TCCTACGGGAGGCAGCAGT | GGACTACCAGGGTATCTAATCCTGTT | J Clin Microbiol 41:4796-4798. | 14532224 |
| hGAPDH | CAACTACATGGTTTACATGTTC | CTCGCTCCTGGAAGATG | [Antiviral Res. 2005 Aug;67(2):76-82.](https://www.ncbi.nlm.nih.gov/pubmed/?term=Bourne+N%2C+Pyles+RB%2C+Yi+M%2C+Veselenak+RL%2C+Davis+MM%2C+et+al.+%282005%29+Screening+for+hepatitis+C+virus+antiviral+activity+with+a+cell-based+secreted+alkaline+phosphatase+reporter+replicon+system.+Antiviral+Res+67%3A+76-82.) | 15927278 |

**Supplemental Table 1.** Primers, references and details of the custom NMB qPCR array. The thermocycling protocol was previously described (Veselenak et al, 2015.) with slight modification as follows: 8 cycle amplification of 95°C for 30 seconds, 48°C for 30 seconds, and 72°C for 30 seconds followed by a 40 cycle amplification of 95°C for 15 seconds, 56°C for 20 seconds and 72°C for 20 seconds during which real time data were acquired at the annealing step . Bio-Rad CFX Manager software was used to complete Cq analysis with a constant baseline adjustment of 50 relative fluorescent units for all array runs. Melt temperatures (Tm) for each amplicon were compared to historical values with an acceptable range of ±0.8°C prior to data analysis.
